# *Ginkgo biloba* Extract Preventively Intervenes in Citrobacter Rodentium-Induced Colitis in Mice

**DOI:** 10.3390/nu15082008

**Published:** 2023-04-21

**Authors:** Tingting Chen, Yiqiang Chen, Kaiyuan Li, Zhuo Chen, Qingyu Zhao, Yimeng Fan, Ying Liu, Suxia Zhang, Zhihui Hao

**Affiliations:** 1 College of Veterinary Medicine, China Agricultural University, Beijing 100193, China; 2 Key Biology Laboratory of Chinese Veterinary Medicine, Ministry of Agriculture and Rural Affairs, Beijing 100193, China; 3 National Center of Technology Innovation for Medicinal Function of Food, National Food and Strategic Reserves Administration, Beijing 100193, China; 4 State Key Laboratory of Animal Nutrition, College of Animal Science and Technology, China Agricultural University, Beijing 100193, China

**Keywords:** *Ginkgo biloba* extract, colitis, Citrobacter Rodentium, gut microbiota, short chain fatty acids

## Abstract

Inflammatory bowel disease (IBD) represents a highly recurrent gastrointestinal disorder and global public health issue. However, it lacks effective and safe strategies for its control. Although *Ginkgo biloba* extract (GBE) has been suggested to exhibit preventive and therapeutic activity for the control of IBD, whether its activity is associated with its ability to modulate intestinal microbiota remains to be addressed. To investigate the effect of GBE on controlling IBD, a Citrobacter Rodentium (CR)-induced mouse colitis model was used, and then histopathological examinations, biochemical assays, immunohistochemistry, and immunoblotting were performed to detect histological changes, cytokines, and tight junction (TJ) proteins in the intestine samples. We also studied 16s rRNA to detect changes in intestinal microbiota and used GC-MS to determine the microbiota-related metabolites short chain fatty acids (SCFAs). The results of our studies revealed that pre-treatment with GBE was sufficient for protecting the animals from CR-induced colitis. As a mechanism for GBE activity, GBE treatment was able to modulate the intestinal microbiota and increase the SCFAs capable of decreasing the pro-inflammatory factors and up-regulating the anti-inflammatory factors while elevating the intestinal-barrier-associated proteins to maintain the integrity of the intestines. Accordingly, our results led to a strong suggestion that GBE should be seriously considered in the preventive control of CR-induced colitis and in the development of effective and safe therapeutic strategies for controlling IBD.

## 1. Introduction

Inflammatory bowel disease (IBD) represents a highly recurrent chronic gastrointestinal disease, and IBD is currently a recognized global public health issue [1,2]. Two main subtypes of IBD are Crohn’s disease (CD) and ulcerative colitis (UC) [3]. Since 2017, CD and UC case counts have reached more than 780,000 and 910,000, respectively, in the United States [4]. IBD incidence can also be high in developing countries. Its long-term inflammation may lead to a higher colorectal cancer risk among patients [3]. Further, the high morbidity of IBD and its associated mortality have become serious concerns and economic burdens to patients and health care systems. Although corticosteroids, anti-inflammatory drugs, and immunosuppressants have been used to treat IBD for more than 10 years [5], they come with drug-associated side effects, such as increased susceptibility to immune tolerance and secondary infection [6]. Thus, developing effective and safe strategies to control IBD is urgently needed.

IBD occurrence has been suggested to be related to intestinal microbial disorders [7,8,9]. The metabolic pathway encoded via the gut microbiome generates numerous host-interacting biologically active molecules, including typical short chain fatty acids (SCFAs) [10]. SCFAs have critical effects on maintaining the intestinal barrier and protective immunity and reducing inflammation [11,12,13]. SCFAs promote tight junction (TJ) (especially ZO-1, Claudin, and Occludin) levels by inhibiting HDAC; meanwhile, SCFAs stimulate the expression of MUC2 through the selective acetylation/methylation of MUC2 [14,15,16]. SCFAs are also able to inhibit pro-inflammatory factors such as TNF-α, IL-6, and IL-1β by inhibiting the HDAC pathway or activating the GPCR41/43 pathway while enhancing anti-inflammatory factor (including IL-10 and IL-4) generation in intestinal immune cells [17,18]. Accordingly, advancing our knowledge of gut microbiota can help develop an effective strategy for the control of IBD.

Citrobacter Rodentium (CR) is a specific extracellular intestinal bacterial pathogen in mice [19]. CR utilizes a Type 3 secretion system (T3SS) to induce the proliferation of colonic crypts and mucosal cells, leading to the excessive growth of CR and changes in gut microbial abundance and diversity within mice [19,20]. CR-mediated epithelial damages in mice share pathogenic characteristics with IBD in humans [19,20,21]. CR-mediated colitis in mice, such as that in C57BL/6 mice, has been well-used as animal models for investigating the pathogenic mechanisms underlying enteric-infection-mediated inflammation [22,23]. Thus, studying CR-induced colitis in C57BL/6 mice was conceivable for advancing our knowledge of gut microbiota in order to develop an effective strategy for controlling IBD.

*Ginkgo biloba* represents a valuable medicinal plant whose leaves and seeds have been considered as additives for food, health, and supplements [24,25,26]. According to the *Essentials of the Materia Medica, Ben Cao Bei Yao*, ginkgo leaf powders blended with flour can be applied to treat intestinal disorders and inflammation. Fresh *Ginkgo biloba* leaves are prepared into ginkgo powder, health care nuts, and drinks to prevent and treat diseases [26]. The main components of *Ginkgo biloba* include flavonoids, polysaccharides, and terpene lactones [24]. The *Ginkgo biloba* extract (GBE) injection GBE761 (Dr. Willmar Schwabe Gmbh & Co. Kg, Baden-wurttemberg, Germany) comprises 24% flavonol glycosides together with 6% terpene lactones, the two components that have been applied in treating cardiovascular and cerebrovascular diseases [27,28,29]. Recently, Yan-Hong Zhou reported that GBE ameliorates rat inflammatory injury within TNBS-mediated rat colitis models through regulating inflammatory factors (SOD, MDA, TNF-α, NF-κBp65, and IL-6) and antioxidation [30]. Venkata showed that EGb761 suppresses macrophage activation, which is also responsible for preventing and treating colitis in mice [31]. Mustafa reported that GBE may be effective in treating UC in rats resulting from acetic acid provided via intracolonic administration through its scavenging activity to deplete oxygen-derived free radicals [32]. However, it is unclear whether the activity of GBE in preventing and treating colitis is associated with its ability to modulate intestinal microbiota and bacterial metabolites.

In this study, we used an animal model and demonstrated the protective ability of GBE to intervene in CR-induced colitis, and we clarified its activity in mice. We also investigated the mechanism for GBE’s ability, through modulation of the gut microbiota, microbiota-released SCFAs, and host cytokines, to intervene in CR-induced IBD.

## 2. Materials and Methods

### 2.1. GBE Synthesis and Identification

Dried *Ginkgo biloba* leaves (Bozhou Haosheng Pharmaceutical Sales Co., Ltd., Bozhou, China) were crushed into coarse powder (10–30 mesh) and extracted twice with 60% ethanol at 65 ± 5 °C, followed by filtration with an 80-mesh screen. The filtrate was mixed with macroporous resin (Shanghai yuanye Bio-Technology Co., Ltd., Shanghai, China) before being eluted with 50% ethanol. The eluent was concentrated via a concentrator (Shanghai Yarong Biochemical Instrument Factory, Shanghai, China) into sedimentary extracts, vacuum freeze-dried, crushed, and passed through an 80-mesh filter for obtaining a crude GBE extract.

Q Exactive Plus-UPLC (Thermo Fisher, Waltham, MA, USA) with Compound Discoverer 3.0 software (Thermo Scientific, Waltham, MA, USA) was later used to analyze the crude GBE extract. The individual components were identified by their retention time, molecular weight (mass deviation of <10 ppm), and MS2 fragment ions with the mzCloud online database and mzVault local database. The components of the flavonol glycosides, terpene lactones, and ginkgolic acid were quantitatively determined using the standard of GBE in the *Chinese Pharmacopoeia 2020*.

### 2.2. Animals

Sixty 6-week-old male C57BL/6 mice (SPF Biotechnology Co., Ltd., Beijing, China) were raised under the following conditions: 23 ± 1 °C, 60 ± 5% humidity, and 12-h/12-h light/dark cycle at the university laboratory animal room (CAU, Beijing, China) with free access to food and water [33]. Our animal study protocols received approval from the Committee of Animal Use and Protection of China Agriculture University (AW11112202-2-2).

### 2.3. Disease Activity Index (DAI)

DAI scores (Table 1) were calculated to determine the colitis level, which included body weight loss, stool consistency, and individual health status. 

### 2.4. Histological Examination

During the necropsy of the mice, tissues were isolated prior to 48–72-h fixation within 10% formalin. After dehydration within alcohols and xylene, the tissues were subjected to paraffin embedding and sectioning in 4-µm slices. After dewaxing of the tissue slices, hematoxylin-eosin (H&E) was added for staining, and then they were dehydrated and sealed for observation under a microscope [33]. For the goblet cell analysis, alcian blue Periodic Acid-Schiff (AB-PAS) was introduced to stain the tissue sections. Tissue histological examinations were performed using Fiji software (NIH, Bethesda, MD, USA).

### 2.5. Immunohistochemistry (IHC)

The tissue sections were dewaxed, the antigens were repaired with citric acid, and 3% hydrogen peroxide was added to block the endogenous peroxidase. Afterward, 10% goat serum was added to block the tissue sections for a 30-min period, followed by overnight incubation using a primary antibody against MUC2 (ab27692, Abcam, Cambridge, UK) and ZO1 (ab276131, Abcam, Cambridge, UK) at 4 °C, after which they were incubated using secondary antibody (K5007, DAKO, Copenhagen, Denmark) for 50 min at an ambient temperature. Color development was performed with 3,3′-diaminobenzidine (DAB), and then they were stained with hematoxylin, dehydrated, and sealed for microscopic examination.

### 2.6. Measurement of Cytokines, Diamine Oxidase (DAO), and D-Lactic Acid (D-LA)

ELISA kits were utilized to determine the contents of factors including IL-1β, TNF-α, IL-6, IL-17A, IL-4, and IL-10. Specifically, the DAO and D-LA contents were determined by an ELISA kit or a biochemical assay kit. These kits were provided by Elabscience Biotechnology Co., Ltd. (Wuhan, China). 

### 2.7. Immunoblotting

After homogenization of the animal tissues, the tissue proteins were extracted with a Total Protein Extraction Maxi Kit BC3710 and quantitatively measured with a BCA Protein Assay Kit PC0020 (Beijing Solarbio Science & Technology Co., Ltd., Beijing, China). The protein aliquots were mixed with 5× protein loading buffer and denatured for a 10-min period at 100 °C. Then, they were electrophoresed on 12% SDS-PAGE, followed by transfer onto 0.45 µm PVDF membranes. After blocking for 2 h using 5% defatted milk at an ambient temperature, the membranes were subjected to overnight incubation using antibodies specific to Occludin (13409-1-AP, Proteintech Group, Inc., Wuhan, China), Claudin1 (13050-1-AP, Proteintech Group Inc., Wuhan, China), Claudin2 (ab53032, Abcam, Cambridge, UK), or Claudin3 (YT0949, Immunoway Biotechnology Co., Ltd, Plano, TX, USA) at 4 °C, and then they were incubated for 2 h using a secondary antibody (HA1006, Hangzhou HuaAn Biotechnology Co., Ltd., Hangzhou, China) at room temperature. A Western Chemiluminescent HRP Substrate kit (Sigma-Aldrich, St. Louis, MO, USA) with an ImageQuant 800 system (GE Healthcare, Wilmington, DE, USA) was utilized to detect the protein blots and Image J software (NIH, Bethesda, MD, USA) was employed for quantification of the protein expression.

### 2.8. 16S rRNA Gene Sequencing

The CTAB/SDS approach was utilized to extract the total genomic DNA in the cecum contents. By using primers (16S515F-806R), the 16S rRNA gene was subjected to amplification in the V3–V4 regions with a Phusion^®^ High-Fidelity PCR Master Mix (Thermo Fisher, MA, USA). Thereafter, the PCR products were detected by electrophoresis on 2% agarose gel, followed by purification using a Qiagen Gel Extraction Kit (Qiagen, Dusseldorf, Germany). A TruSeq^®^ DNA PCR-Free Sample Preparation Kit (Illumina, San Diego, CA, USA) was used for generating the sequencing libraries, and an Agilent Bioanalyzer 2100 system and Qubit@2.0 Fluorometer (Thermo Fisher, Waltham, MA, USA) were applied for the assessments. Then, the Illumina NovaSeq platform was used for the library sequencing to generate the 250-bp paired-end reads.

The alpha analyses, including the Chao1, Simpson, and Shannon_2 indices, were conducted based on USEARCH (version 11.2.64). The Vegan Package (Version 2.4.2) in R was used to generate the principal coordinates analysis (PCoA) and non-metric multidimensional scaling (NMDS) figures, and then we conducted the Anosim analysis. LEfSe (version 1.0.8) was employed for the linear discriminant analysis (LDA score of ≥2). The raw reads count tables at the phylum and genus taxonomic levels were used for the differential abundance analysis with DEseq2.

### 2.9. Measurement of SCFAs

Twenty mg of colon contents were mixed with 800 µL of 0.5% phosphoric acid water (containing 10 µg/mL of 2-ethylbutyric acid as the endogenous reference) and ground at 4 °C. The mixture was then ultrasonically extracted for a 10-min period, followed by 15 min of centrifugation at 13,000× *g*, also at 4 °C. The supernatants (200 µL) were added to 200 µL of n-butanol, ground, ultrasonically extracted at 4 °C for a 10-min period, and then centrifuged for 15 min at 13,000× *g,* again at 4 °C. We utilized 8890B-5977B GC/MSD (Agilent Technologies Inc., Santa Clara, CA, USA) for the supernatant analysis. The data were processed using Masshunter quantitative software (Agilent Technologies Inc., Santa Clara, CA, USA, version: v10.0.707.0). The GC-MS detection conditions can be observed in Table S1.

### 2.10. Statistical Analyses

GraphPad Prism 8.0 (GraphPad Software, San Diego, CA, USA) was employed for the statistical analysis of the results, which were represented by means ± SDs. One-way ANOVA was used to compare two groups, while the LSD method was adopted to compare several groups. A *p* value of <0.05 stood for significant differences [34].

## 3. Results

### 3.1. Preparation and Identification of GBE

**Figure 1:**
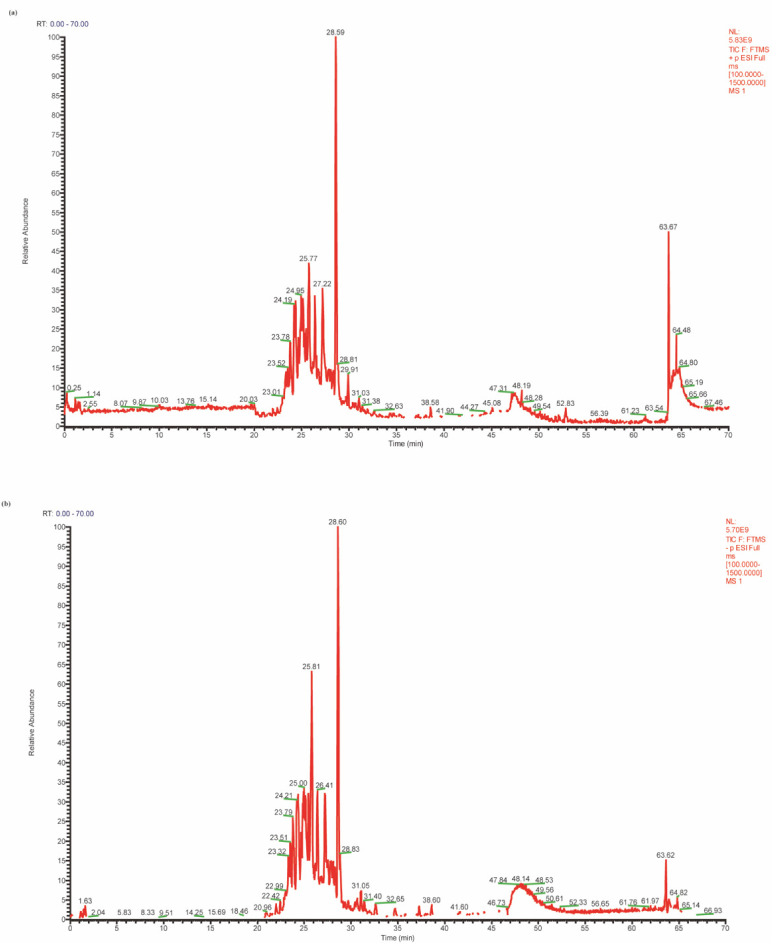
TIC diagram of the GBE in the positive (**a**) and negative (**b**) ion modes.

To verify the quality of our prepared GBE in compliance with the standard set by the *Chinese Pharmacopoeia 2020*, we used high-resolution mass spectrometry and HPLC techniques to qualitatively identify and quantitatively determine the GBE’s components. Using Q Active Orbitrap high-resolution mass spectrometry in positive and negative ion modes, we generated a TIC chromatogram of the *Ginkgo biloba* extract (Figure 1). Screening and analysis of the detected compounds revealed that the GBE mainly consisted of 62 chemical components, including 43 flavonoids, 6 Terpenoids, 2 alkylphenylenic acids, 7 carboxylic acids, and 4 lignans (Table S2). The flavonoids included 21 Flavonol and its glycosides, 10 Flavone and its glycosides, 5 Flavanone and its glycosides, 3 flavon-3-ols, and 4 biflavonoids. The contents of flavonol glycosides, ginkgolides, and ginkgolic acid were further evaluated with the standard method in the *Chinese Pharmacopoeia 2020*. The results showed that the contents of flavonol glycosides, terpene lactones, and ginkgolic acid were 24.57% (≥24%), 6.09% (≥6%), and 3.27 mg/kg (≤5 mg/kg), respectively; these components were all in accordance with the pharmacopoeia standards to be used in the following studies.

### 3.2. GBE Ameliorated Symptoms of CR-Induced Colitis in Mice

**Figure 2:**
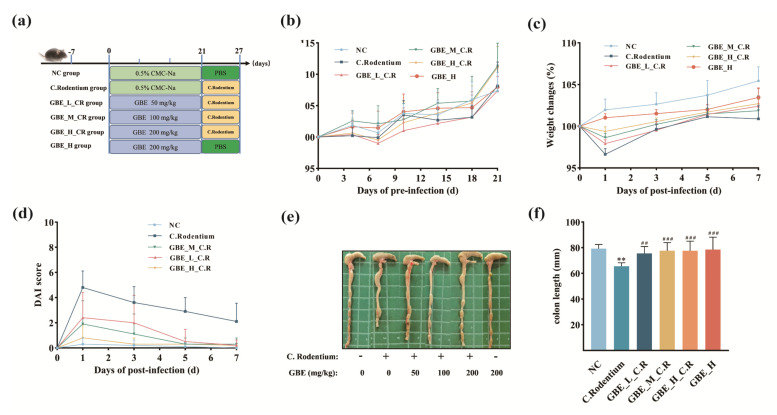
GBE ameliorated the CR-mediated colitis symptoms in mice. (**a**) Experimental design grouping and treatment (n = 10 per group). (**b**) BW changes before CR infection. (**c**) BW changes after CR infection. (**d**) DAI scores. (**e**,**f**) Colon lengths. ** *p* < 0.01 vs. NC group; ### *p* < 0.001 an ## *p* < 0.01 vs. C. Rodentium group.

For investigating if GBE protected animals from CR-induced colitis, 60 mice were randomized into six groups (n = 10) (Figure 2a). GBE was dissolved in the vehicle buffer of 0.5% carboxymethylcellulose sodium (CMC-Na). The mice were orally administered CMC-Na or GBE at different concentrations (50 mg/ kg, GBE-L, n = 10; 100 mg/ kg, GBE-M, n = 10; and 200 mg/kg, GBE-H, n = 20) once per day for 21 days. On day 22, the mice were gavaged with saline or 2 × 10^9^ CFU/mouse of CR (DBS 100, ATCC 51459) [35]. After 8 days of infection with CR, blood, colonic samples, colonic contents, and cecal contents, together with the major organs, were harvested and preserved at −80 °C prior to subsequent analysis [33].

As shown in Figure 2b, the body weights (BWs) of the animals in all the groups increased, and the mice supplemented with GBE gained more BW than the control NC group. GBE treatment significantly ameliorated the CR-induced weight loss (Figure 2c), colon shortening (Figure 2e,f), and DAI index (Figure 2d) in a dose-dependent manner. Additionally, the organ indexes (Figure S1) and histological evaluations (Figure S2) showed that there were no adverse effects of the GBE on the hearts, livers, spleens, lungs, and kidneys of the mice resulting from the administration of 200 mg/kg of GBE. The results indicated that GBE supplementation promoted the growth of mice and ameliorated the CR-induced colitis.

### 3.3. GBE Improved Colonic Lesions in Mice

**Figure 3:**
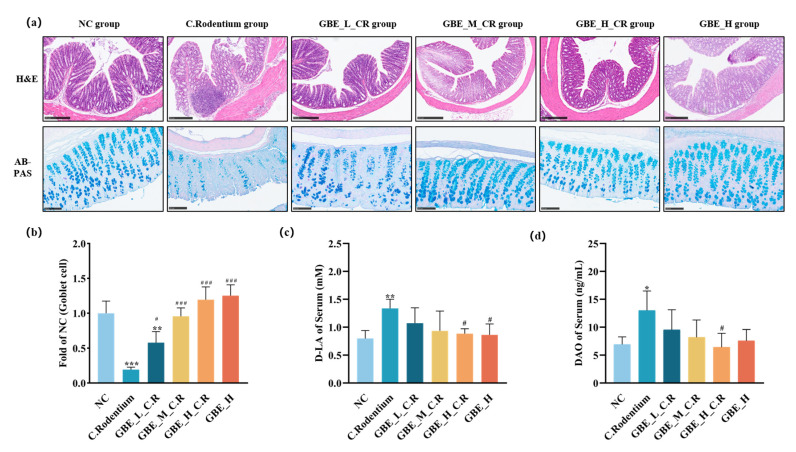
GBE improved the colonic lesions in mice. (**a**) Typical images showing H&E staining on colonic sections (scale bar = 250 µm). Typical images showing AB-PAS staining on colonic sections (scale bar = 100 µm). (**b**) Goblet cell positive density. (**c**) Serum D-LA contents. (**d**) Serum DAO contents. *** *p* < 0.001, ** *p* < 0.01, and * *p* < 0.05 vs. NC group; ### *p* < 0.001 and # *p* < 0.05 vs. C. Rodentium group.

Transmissible murine colonic hyperplasia represents a hallmark of CR-induced symptoms and is characterized by the growth of epithelial cells, thickening of the mucosa, and hyperplasia of the colonic crypt [36]. To understand how GBE affected the CR-mediated colitis, H&E and AB-PAS staining was conducted on the colon tissues. The H&E staining (Figure 3a) revealed significant pathological changes induced by CR, including colon thickening, infiltration of inflammatory cells, and loss of goblet cells. In contrast, those pathological symptoms were significantly reduced by GBE, and the goblet cells were significantly protected by GBE (dose-dependently) (*p* < 0.01) (Figure 3a,b). To further investigate how GBE affected intestinal permeability, we measured the serum DAO and D-LA contents. As shown in Figure 3c,d, GBE did not induce any changes in the D-LA and DAO contents; however, GBE treatment significantly reversed the CR-increased D-LA and DAO contents (*p* < 0.05). Accordingly, the results indicated that GBE supplementation was able to protect animals from CR-induced pathological symptoms, goblet cell loss, and intestinal permeability.

### 3.4. GBE Suppressed the Pro-Inflammatory Factors and Promoted the Anti-Inflammatory Factors within Mice

**Figure 4:**
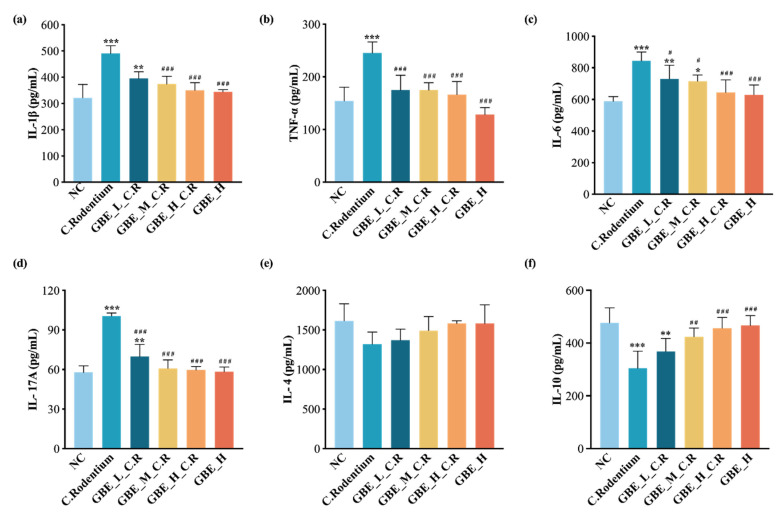
GBE suppressed the pro-inflammatory factors IL-1β (**a**), TNF-α (**b**), IL-6 (**c**), and IL-17A (**d**), whereas it increased the anti-inflammatory factors IL-4 (**e**) and IL-10 (**f**) in the mice. All the inflammatory factor contents were determined using the ELISA kits. *** *p* < 0.001, ** *p* < 0.01, and * *p* < 0.05 vs. NC group; ### *p* < 0.001, ## *p* < 0.01, and # *p* < 0.05 vs. C. Rodentium group.

Cytokines have critical effects on inflammatory responses to pathogenic infections [37]. The colonic cytokine contents were analyzed, and it was detected that CR infection promoted the pro-inflammatory factors IL-1β, TNF-α, IL-6, and IL-17A (Figure 4a–d) (*p* < 0.01), as well as the anti-inflammatory factors IL-4 (*p* > 0.05) and IL-10 (*p* < 0.01) (Figure 4e,f). As expected, GBE treatment reduced CR-induced pro-inflammatory cytokines and promoted IL-4 and IL-10 contents (Figure 4e,f). Additionally, the CR-induced changes in the IL-1β, TNF-α, IL-6, IL-17A, and IL-10 levels of the mice were remarkably reversed by GBE (Figure 4a–d,f), though GBE treatment did not significantly reverse the IL-4 levels (Figure 4e). Thus, GBE treatment was able to improve CR-induced intestinal inflammation through both reducing the pro-inflammatory factors and promoting the anti-inflammatory cytokine levels of the mice with colitis.

### 3.5. GBE Enhanced Mouse Intestinal Barrier Function

**Figure 5:**
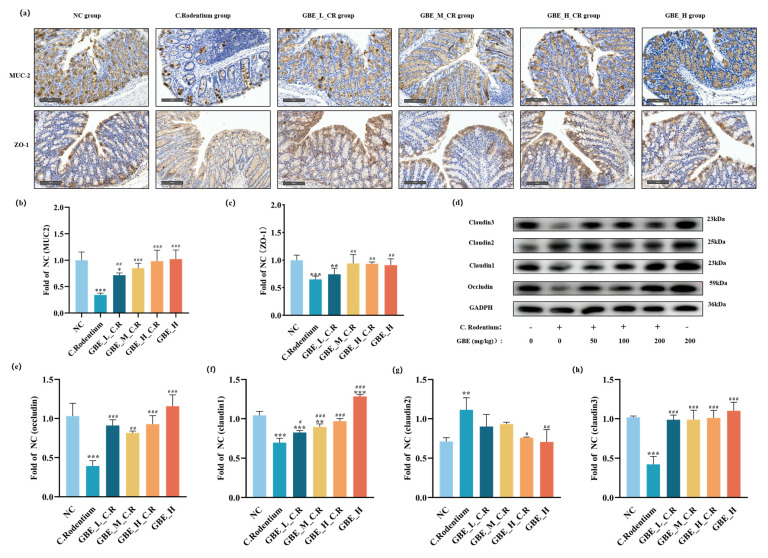
GBE enhanced mouse intestinal barrier function. (**a**) Typical IHC images showing MUC2 and ZO1 (scale bar = 250 µm). The quantification of MUC2 (**b**) and ZO1 (**c**). (**d**) Typical protein bands for Occludin, Claudin1, Claudin2, and Claudin3. Protein levels of Occludin (**e**), Claudin1 (**f**), Claudin2 (**g**), and Claudin3 (**h**) compared to GAPDH. *** *p* < 0.001, ** *p* < 0.01, and * *p* < 0.05 vs. NC group; ### *p* < 0.001, ## *p* < 0.01, and # *p* < 0.05 vs. C. Rodentium group.

The intestinal barrier has an essential role in maintaining intestinal health [38]. Using IHC methods, we detected that the levels of MUC2 (Figure 5a,b) and ZO1 (Figure 5a,c) were decreased in the mice infected with CR, and these levels were up-regulated after GBE treatment (dose-dependently). Feeding 200 mg/kg GBE also dramatically enhanced MUC2 and ZO1 contents within the healthy mice (Figure 5a–c). The level of TJ proteins was further examined through immunoblotting, and the results showed that Occludin, Claudin1, and Claudin3 (Figure 5d–f,h)) were reduced and Claudin2 (Figure 5d,g) was up-regulated within the CR-mediated colitis mice, and this was reversed with GBE treatment. GBE treatment resulted in dose-dependent increases in Occludin, Claudin1, and Claudin3 (Figure 5d–f,h). GBE treatment also significantly reversed CR-increased Claudin2 (Figure 5g). Thus, GBE treatment was able to improve intestinal barrier function by increasing the intestinal protein levels of MUC2, ZO1, Occludin, Claudin1, and Claudin3, as well as decreasing Claudin2.

### 3.6. GBE Modulated Gut Microbiota

**Figure 6:**
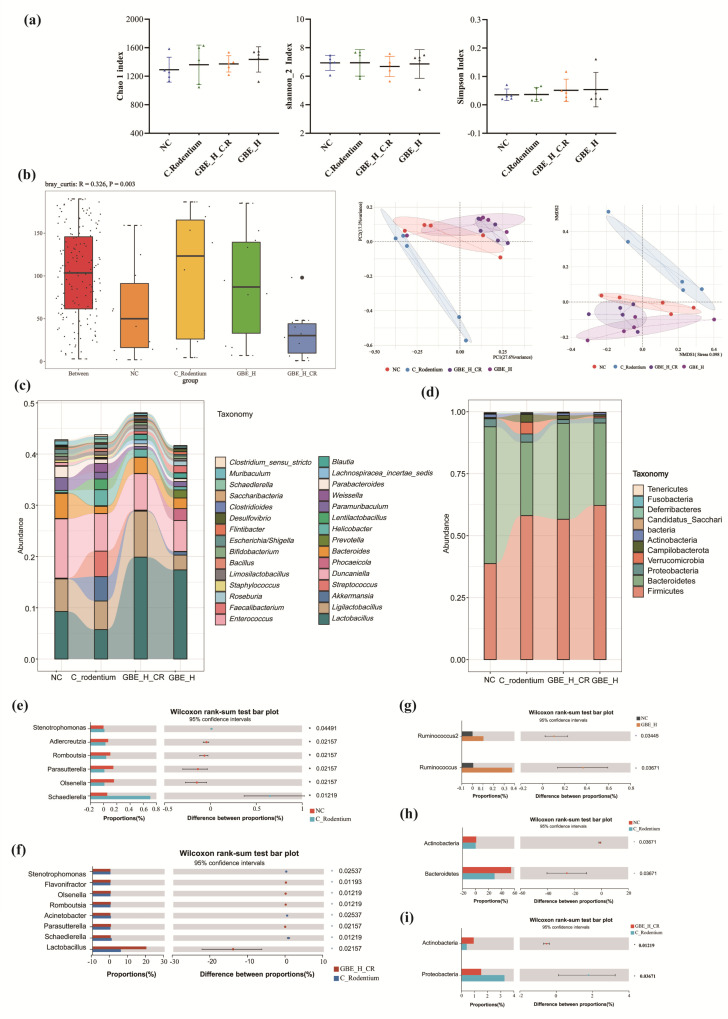
GBE modulated gut microbiota. (**a**) α diversity represented by the Chao 1, Shannon_2, and Simpson indices. (**b**) β diversity under Anosim, NDMS, and PCoA analysis. Taxonomic distributions regarding gut microbial compositions at the genus (**c**) and phylum (**d**) levels. Wilcoxon rank-sum test for the NC and C. Rodentium groups at the genus level (**e**), the GBE_H_CR and C. Rodentium groups at the genus level (**f**), the NC and GBE_H groups at the phylum level (**g**), the NC and C. Rodentium groups at the phylum level (**h**), and the GBE_H_CR and C. Rodentium groups at the phylum level (**i**).

Intestinal microbial dysbiosis is an important contributing factor to IBD [39]. To detect how the GBE supplementation affected the intestinal flora of the mice with colitis, 16S rRNA sequencing was performed for investigating the intestinal microbial composition of the animals infected with CR and treated with GBE. The results of the alpha diversity assay did not reveal any significant changes in the Chao 1, Shannon_2 and Simpson indices in the mice infected with CR or treated with GBE (Figure 6a). Interestingly, Anosim, NDMS, and PCoA analysis revealed significant clustering changes in the microbiota composition resulting from CR infection (Anosim = 0.003). EBG alone induced slight changes (GBE vs. control) in the microbial composition, and EBG reversed the CR-induced changes (Figure 6b). Thus, the results indicated that GBE protected the animals from the CR-induced changes in the intestinal microbiota, and it also affected the normal flora.

Further analyses of the effect of GBE on the gut microbiota at both the phylum (Figure 6c) and genus (Figure 6d) levels revealed that the cecal flora at the phylum level included primarily *Firmicutes*, *Bacteroidetes*, and *Proteobacteria,* with a relative abundance of 90% (Figure 6d). CR infection resulted in reducing the abundance of *Bacteroidetes* and Actinobacteria (Figure 6h), and GBE treatment alleviated the CR-induced changes, especially for Actinobacteria (*p* < 0.05) (Figure 6i). Additionally, GBE administration did not significantly change the gut microbiota within the healthy mice. CR infection led to elevated *Schaedlerella* and *Stenotrophomonas* abundances and decreased *Adlercreutzia*, *Olsenella*, *Parasutterella*, and *Romboutsia* abundances at the genus level (Figure 6e), and the GBE treatment alleviated these changes, particularly the *Schaedlerella*, *Stenotrophomonas*, *Olsenella*, *Parasutterella*, and *Romboutsia* abundances (*p* < 0.05) (Figure 6c,f). Besides, GBE intervention greatly promoted *Flavonifractor* and *Lactobacillus* abundances (*p* < 0.05) (Figure 6f). In addition, GBE treatment enhanced *Ruminococcus* and *Ruminococcus2* abundances in the healthy mice (Figure 6g).

**Figure 7:**
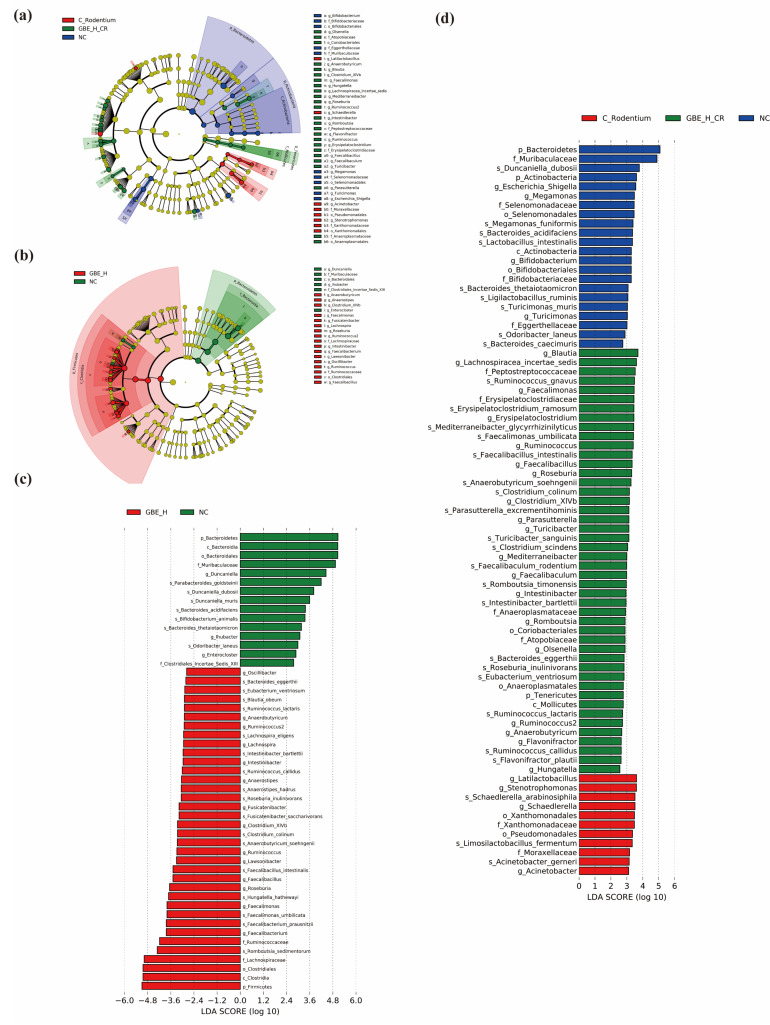
Role of GBE supplementation in key microbial phylotypes. Taxonomic cladogram (**a**) and histogram of LDA scores (**d**) obtained by LEfSe analysis among the NC, C. Rodentium, and GBE_H_CR groups. Taxonomic cladogram (**b**) and histogram of LDA scores (**c**) obtained by LEfSe analysis between the NC and GBE_H groups.

We further performed LEfSe analysis (LDA of >2, *p* < 0.05) of the bacterial phenotypic variation at the phylogenetic level from phylum to genus. We detected *Bacteroidetes*, *Muribaculaceae*, *Duncaniella*, and *Actinobacteria* in the healthy mice and *Stenotrophomonas*, *Schaedlerella*, and *Acinetobacter* in the mice infected with CR. *Olsenella*, *Tenericutes*, *Parasutterella*, *Romboutsia*, *Flavonifractor*, and *Turinobacteria* were found in the mice infected with CR and treated with GBE (Figure 7a,d). The most abundant flora were the phylum *Firmicutes*, family *Lachnospiraceae*, family *Ruminococcaceae*, and genus *Ruminococcus* and *Ruminococcus2* in the healthy mice treated with GBE, and the most abundant flora were the phylum *Bacteroidetes*, family *Muribaculaceae*, and the genus *Duncaniella* in the healthy mice not treated with GBE (Figure 7b,c). Accordingly, GBE treatment regulated gut microbiota and exerted a protective effect on the mice with colitis.

### 3.7. GBE Promoted Intestinal SCFAs in Mice

**Figure 8:**
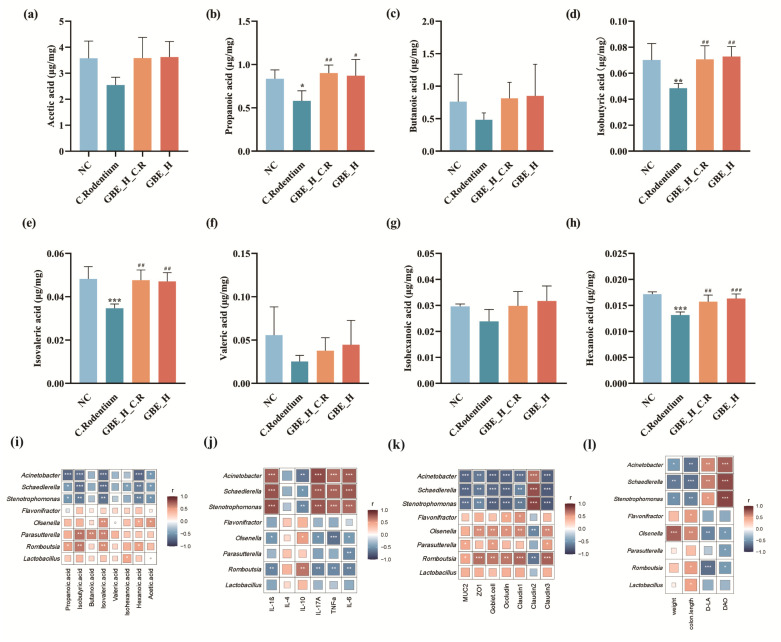
Mean SCFAs levels within the mouse colonic contents and Spearman’s correlation analysis. (**a**) Acetic acid. (**b**) Propanoic acid. (**c**) Isobutyric acid. (**d**) Butanoic acid. (**e**) Isovaleric acid. (**f**) Valeric acid. (**g**) Isohexanoic acid. (**h**) Hexanoic acid. Spearman’s correlation analysis of changes between abundances of individual genera and (**i**) SCFAs; (**j**) cytokines; (**k**) TJ, MUC2, ZO1, and goblet cell levels; and (**l**) weight, colon length, and D-LA and DAO levels. *** *p* < 0.001, ** *p* < 0.01, and * *p* < 0.05 vs. NC group; ### *p* < 0.001, ## *p* < 0.01, and # *p* < 0.05 vs. C. Rodentium group.

SCFAs, the intestinal flora metabolites, have critical effects on maintaining intestinal barrier function and anti-inflammation [36]. We investigated how GBE affected eight colonic SCFAs using GC-MS. We detected that the eight SCFAs, acetic acid, propanoic acid, isobutyric acid, butanoic acid, isovaleric acid, valeric acid, isohexanoic acid, and hexanoic acid, were reduced in the colons of the mice infected with CR, whereas GBE treatment reversed the CR-induced reduction of these SCFAs (Figure 8a–h). Hexanoic acid, isobutyric acid, isovaleric acid, and hexanoic acid exhibited significant differences (*p* < 0.05). These SCFAs were not changed by GBE treatment. Thus, GBE treatment appeared to be able to prevent the bacteria-produced SCFAs from the CR-induced reductions in the colon.

### 3.8. Correlation Analysis of Gut Microbiota and SCFAs, Cytokines, and TJ Proteins

To further understand the potential effects of bacterial SCFAs on the host cytokines and TJ proteins, we performed Spearman’s correlation analysis, and we detected that at the genus level, the intestinal microbiota *Acinetobacter*, *Schaedlerella* and *Stenotrophomonas* were positively correlated with the pro-inflammatory cytokines IL-1β, IL-17A, TNF-α, and IL-6 (Figure 8i), as well as the Claudin2, D-LA and DAO (*p* < 0.01) (Figure 8k,l), but negatively correlated with IL-10 (Figure 8j), acetic acid, propanoic acid, isobutyric acid, isovaleric acid, and hexanoic acid (Figure 8j), as well as Occludin, Claudin1, Claudin3, MUC2, ZO1, and goblet cells (*p* < 0.01) (Figure 8k); however, *Flavonifractor*, *Olsenella*, *Parasutterella* and *Romboutsia* were in the opposite correlation (Figure 8i–l). Thus, CR infection reduced the content of beneficial bacteria *Flavonifractor*, *Olsenella*, *Parasutterella* and *Romboutsia* while promoting harmful bacteria *Acinetobacter*, *Schaedlerella* and *Stenotrophomonas*, resulting in reducing SCFAs, pro-inflammatory cytokines, intestinal permeability, and barrier that were effectively inhibited by GBE treatment.

## 4. Discussion

This work was the first to demonstrate that GBE effectively protected mice from CR-induced IBD. In our IBD animal model, orally infecting the C57BL/6 mice with CR resulted in IBD-associated symptoms, including animal body weight loss, shortened colons, thickened colon walls, and colonic inflammation. In addition, CR infection induced gut microbial dysbiosis, decreased microbiota-associated metabolite SCFAs, caused intestinal inflammation, and disrupted intestinal barrier integrity. The oral administration of GBE by gavage was sufficient to modulate gut microbiota populations, SCFAs, host cytokines, and TJ proteins. Pre-treatment with GBE effectively protected animals from CR-induced IBD and changes in gut microbiota, SCFAs, host cytokines, and TJ proteins. Our findings strongly suggested that GBE was able to intervene in CR-induced IBD. 

IBD is related to gut dysbiosis [8,9]. Over 95% of gut bacteria are of the phyla *Bacteroidetes*, *Firmicutes*, *Proteobacteria*, or *Actinobacteria*. GBE intervention significantly altered the diversity of gut microbiota induced by CR infection, which was consistent with the findings of Zhang Yunchang and Feng Yu [35,40]. According to analysis at the phylum level, 200 mg/kg of GBE administered orally in mice enhanced intestinal *Bacteroidetes* and *Actinobacteria* abundances in CR-infected mice while decreasing *Proteobacteria* abundance. Based on further analysis of changes in genus-level bacterial communities, GBE enhanced SCFA-producing bacterial abundances, such as *Flavonifractor*, *Lactobacillus*, *Romboutsia*, or *Olsenella*, which was consistent with previous reports of decreased levels of these bacterial populations in mice with colitis [2,41,42,43]. Dietary supplements such as rice bran have been shown to significantly enrich *Flavonifractor* in the feces of colon cancer patients [44]. Ayane Mikami found that *Flavonifractor* is able to help reduce intestinal inflammation in DSS-mediated colitis mice through inhibiting IL-17A [45]. *Olsenella* has been shown to support the treatment of colon cancer through the microbial metabolite immune pathway [46]. It has been shown that dietary supplementation with dendrobium fimbriatum hook polysaccharide or phloretin results in reduced populations of *Acinetobacter* and *Stenotrophomonas* [2,47]. Our study revealed that GBE significantly reduced the abundances of the conditional pathogenic bacteria *Acinetobacter*, *Schaedlerella*, and *Stenotrophomonas* in the colons of mice, which may have contributed to the ability of GBE to alleviate CR-induced changes in gut microbiota in the pathogenesis of IBD. Thus, GBE treatment maintained the intestinal microenvironment through elevating beneficial bacterial quantities while decreasing pathogenic bacterial quantities within the IBD mice.

SCFAs, generated via gut microbiota, have critical effects on colon health and integrity [48]. It has been shown that increases in intestinal SCFAs, including acetic acid, propanoic acid, isobutyric acid, or isovaleric acid, by dietary supplementation with probiotics or plant extracts contributes to the control of colitis [49,50]. The phyla Bacteroidetes and *Actinobacteria* and the genera *Flavonifractor*, *Lactobacillus*, *Romboutsia*, and *Olsenella* are proposed to be beneficial for the production of intestinal SCFAs, in particular, acetic acid, propionic acid, butyric acid, and valeric acid [42,51,52]. Our study revealed that pre-treating animals with 200 mg/kg GBE increased acetic acid, propanoic acid, isobutyric acid, butanoic acid, isovaleric acid, valeric acid, isohexanoic acid, and hexanoic acid levels within the colon in association with increased abundances of Bacteroidetes, *Flavonifractor*, *Romboutsia*, and *Olsenella*. Thus, GBE significantly promoted the SCFAs produced by the bacteria *Bacteroidetes*, *Actinobacteria*, *Flavonifractor*, *Lactobacillus*, *Romboutsia*, and *Olsenella* to maintain intestinal health and intervene in the CR-induced colitis.

SCFAs have been shown to modulate cytokine generation [53] while increasing TJ protein levels in the intestine [54] to alleviate IBD. Studies have shown that acetic acid, propionic acid, and butyric acid relieve gut inflammation through inhibiting the pro-inflammatory factor levels IL-6, IL-1β, and TNF-α and promoting anti-inflammatory cytokine generation, such as IL-10 and IL-4 [17,18,55]. In addition, acetic acid can restore the decreased expression of MUC2 proteins in colonic crypts and repair the damaged mucus barrier [56], and butyric acid can alleviate colitis through enhancing IL-22 production [57]. Propionic acid [58] and butyric acid [49] can regulate TJ protein levels, especially ZO-1, Claudin, and Occludin, to maintain intestinal barrier function. Propionic acid can also promote the release of IL-10 by activating GPR41 and GPR43 to alleviate intestinal inflammation [59]. Therefore, GBE treatment inhibited the pro-inflammatory factors IL-1β, IL-6, and TNF-α while promoting the anti-inflammatory factors IL-4 and IL-10, as well as the production of MUC2 and TJ proteins through SCFAs, thereby reducing intestinal inflammation while maintaining intestinal barrier integrity.

Cytokines are key mediators for the interactions between intestinal cells [37]. Intestinal inflammation is related to higher TNF-α, IL-1β, and IL-6 levels [60]. TNF-α represents a multifunctional pro-inflammatory factor that stimulates the generation of other pro-inflammatory factors, such as IL-1 and IL-6 [61]. IL-1β activation accounts for an important link in regulating pro-inflammatory responses, and as a result, secondary inflammatory factors such as IL-6 are activated [62]. The release of inflammatory cytokines into targeted tissues directly promotes the development of inflammation in organs, such as intestines [63]. In our study, IL-1β, TNF-α, IL-6, and IL-17A contents within the colon increased after CR infection, conforming to prior research regarding CR-induced colitis [40,64]. We also detected that colonic IL-17A contents were elevated after CR infection. IL-17A is a hallmark factor secreted by Th17 cells, and it can effectively mediate the inflammatory response during early infection or injury [65]. Elevated levels of IL-17A have been identified in mouse colitis induced by TNBS or CR [66,67]. IL-4 and IL-10 are two anti-inflammatory factors with important roles in inflammation [62]. Our study revealed that their colonic contents decreased in the mice after CR infection, especially IL-10 elevation. IL-10 is critical in controlling infections in the colon as IL-10-deficient mice developed severe and sustained diarrhea and colitis within 1–2 weeks after infection [68]. IL-10 elevation has been shown to alleviate colitis [69]. Our studies revealed that an improvement in colitis was associated with cytokines in a dose-dependent manner in the GBE-treated mice. The healthy mice supplemented with 200 mg/kg of GBE also showed remarkably decreased intestinal TNF-α, IL-6, IL-1β, and IL-17A levels, as well as an increased IL-10 level, which was consistent with other reports [30,31,32]. The main GBE component flavonoids, such as kaempferol [70] and isorhamnetin [50], have been shown to reduce IL-1β, IL-6, and TNF-α within DSS-induced colitis animal models. Quercetin [45] can inhibit pro-inflammatory factor generation, such as IL-17, TNF-α, or IL-6, while promoting IL-10 production during CR-induced colitis. Terpenoid component bilobalide [71] can inhibit the expression of IL-1β, IL-6, and TNF-α in DSS-mediated colitis. Therefore, these flavonoid and terpenoid components of GBE may reduce inflammatory levels in mice with colitis.

The intestinal barrier is prerequisite for maintaining the stability of the intestinal environment, and the intestinal mucous layer and intercellular TJ levels have critical effects on maintaining intestinal barrier integrity [38]. Epithelial cell injury or mucous layer loss can promote the occurrence of colon cancer, spontaneous colitis, and the infection and colonization of the intestinal pathogen CR [72,73]. The colonization of CR in the intestine can induce various intestinal damages, disrupt intestinal barrier function, and increase intestinal permeability [38]. Our results suggested that the CR infection caused colon thickening and inflammatory cell infiltration, reduced goblet cell quantities, and increased serum D-LA and DAO levels. DAO represents the intracellular enzyme within intestinal mucosal epithelial cells, and D-LA is a bacterial fermentation metabolite. Increased serum D-LA and DAO contents reflect the degree of mucosal epithelial cell damage and mucosal permeability in the intestine [74,75]. Our results showed that GBE improved the mouse colon injuries caused by CR, increased the colonic goblet cell quantities, and reduced intestinal permeability. TJ proteins, such as Occludin, Claudin, ZO1, and desmosomes, together with intestinal epithelial cells, form the mechanical barrier of the intestine. Goblet cells and their secreted MUC2 are important components of the intestinal mucosal barrier [72]. In contrast, increased Claudin2 is associated with CR infection, and the overexpression of Claudin 2 in intestinal epithelial cells enhanced colitis in mice [43,76]. Claudin2 plays a contradictory role to Claudin.

Accordingly, our studies demonstrated that pre-treating mice with GBE increased SCFA-generating bacterial abundances, thereby promoting SCFA production. SCFAs suppressed pro-inflammatory cytokines and promoted anti-inflammatory cytokine, resulting in increasing MUC2, ZO-1, Claudin, and Occludin to maintain intestinal barrier function. It is conceivable that the GBE protection against CR-induced colitis in the mice might have been achieved through its flavonoid components, such as quercetin, isorhamnetin, kaempferol, and ginkgolide [66,70,71,77]. However, the underlying mechanisms of these components for protecting mice from CR-induced colitis remains to be determined.

## 5. Conclusions

Our research demonstrated that pre-treating mice with GBE at 200 mg/kg was sufficient to result in an effective and safe protection of the animals from CR-induced colitis, and it alleviated the CR-induced symptoms and modulated the mechanisms underlying the IBD pathogenic mechanism. Our results led to the suggestion that GBE should be seriously considered in the preventive control of CR-induced colitis and in developing anti-IBD treatments with high levels of safety and effectiveness.

## Figures and Tables

**Table 1:** The standard DAI scores.

Score	Weight Loss %	Stool Consistency	Health Status
0	none	normal	normal
1	0–5		
2	6–10	loose stool	poor
3	11–15		
4	>16	diarrhea	terrible

## Data Availability

All data utilized in the present work can be obtained from the corresponding author on request.

## Supplementary Materials

The following supporting information can be downloaded at: https://www.mdpi.com/article/10.3390/nu15082008/s1, Table S1: GC-MS detection conditions; Table S2: Chemical components of GBE; Figure S1: The organ coefficients of the hearts, livers, spleens, lungs, and kidneys (a–e) of all groups; Figure S2: The histological evaluations of the hearts, livers, spleens, lungs, and kidneys of all groups.
