# Supplementary material for: Ginkgo biloba Extract Preventively Intervenes in Citrobacter Rodentium-Induced Colitis in Mice
Source: Nutrients. 2023 Apr 21;15(8):2008. doi: 10.3390/nu15082008 (PMC10145670; doi:10.3390/nu15082008)
Supplement: Supplementary file 1 [file nutrients-15-02008-s001.zip › nutrients-2355261-supplementary.pdf]

Table S1. GC-MS detection conditions.

Chromatographic Conditions	
HP FFAP Capillary Column	30 m × 0.25 mm × 0.25 μm,
	Agilent J&W Scientific, Folsom, CA, USA
Carrier Gas	high purity helium (purity ≥ 99.999%)
Flow Rate	1.0 mL/min
Inlet Temperature	180 ℃
Injection Volume	1 μL
Split Ratio,	10:1
Solvent Delay	2.5 min
Initial Temperature	80 ℃
Increased Temperature 2	120 ℃, 20 ℃/min
Increased Temperature2	160 ℃, 5 ℃/min
Final Temperature	220 ℃, 3 min
Mass spectrometry Conditions	
Electron Bombardment Ion Source	EI
Ion Source Temperature	230 ℃
Quadrupole Temperature	150 ℃
Transmission Line Temperature	230 ℃
Electron Energy	70 eV
Scanning Mode	SIM

Table S2. Chemical components of GBE.

No.	Name	Formula	Calc. MW	RT [min]	mzVault Best Match	Group Area
	Flavonol and its glycosides					
1	Quercetin	C15 H10 O7	302.0421	25.393	92.1	3.45E+09
2	Quercetin-3β-D-glucoside	C21 H20 O12	464.0952	25.190	97.5	3.28E+09
3	Quercitrin	C21 H20 O11	448.1002	26.164	91.4	2.40E+09
4	Isoquercitrin	C21 H20 O12	464.0951	24.728	89.2	7.37E+08
5	Rutin	C27 H30 O16	610.1527	24.744	96.8	9.15E+09
6	Kaempferol	C15 H10 O6	286.0472	26.243	90.1	4.75E+09
7	Kaempferol-3-O-rutinoside	C27 H30 O15	594.158	25.504	91.3	6.30E+09
8	Kaempferol 3-glucorhamnoside	C27 H30 O15	594.1581	26.227	91	6.70E+09
9	Astragalin	C21 H20 O11	448.1001	25.489	90.7	7.86E+08
10	Kaempferol-7-O-glucoside	C21 H20 O11	448.1002	26.806	86.3	1.35E+09
11	Afzelin	C21 H20 O10	432.1052	27.236	87.5	6.06E+08
12	Kaempferol-3-O-β-glucopyranosyl-7-O-α-rhamnopyranoside	C27 H30 O15	594.158	23.65	75.3	1.56E+08
13	Kaempferol-7-O-β-D-glucopyranoside	C21 H20 O11	448.1001	23.355	85.5	3.30E+07
14	Leucoside	C26 H28 O15	580.1426	25.315	76.4	3.73E+07
15	Tiliroside	C30 H26 O13	594.1374	24.538	72	3.00E+07
16	Isorhamnetin	C16 H12 O7	316.0579	26.201	84.9	4.04E+08
17	Isorhamnetin-3-O-nehesperidine	C28 H32 O16	624.1683	24.380	87.7	3.35E+08
18	Typhaneoside	C34 H42 O20	770.2261	24.396	91.3	1.80E+09
19	Syringetin-3-O-glucoside	C23 H24 O13	508.1214	25.710	75.9	7.77E+07
20	Myricetin	C15 H10 O8	318.0373	27.14	92.7	9.38E+08

				0		
21	Grosvenorine	C33 H40 O19	740.2157	24.332	85.7	
	Flavone and its glycosides					
22	Diosmetin	C16 H12 O6	300.063	31.641	85.8	1.42E+08
23	Diosmetin-7-O-β-D-glucopyranoside	C22 H22 O11	462.1159	26.555	77.3	7.83E+08
24	Apigenin-7-O-β-D-glucoside	C21 H20 O10	432.105	26.305	90.9	3.01E+09
25	Apigenin	C15 H10 O5	270.0525	31.196	94.7	5.45E+08
26	Vicenin II	C27 H30 O15	594.1578	23.318	81.4	5.20E+07
27	Genkwanin	C16 H12 O5	284.0682	36.328	87.2	2.04E+08
28	Sakuranetin	C16 H14 O5	286.0837	35.978	81.5	1.46E+08
29	Mearnsitrin	C22 H22 O12	478.1106	24.892	71.7	1.20E+08
30	Chrysin	C15 H10 O4	254.0576	35.839	54.6	4.15E+06
31	Baicalin	C21 H18 O11	446.0847	27.568	79.1	2.44E+07
	Flavanone and its glycosides					
32	Naringenin	C15 H12 O5	272.068	26.367	91.2	3.00E+08
33	4',7-Dihydroxyflavanone	C15 H12 O4	256.073	26.933	59.5	8.42E+07
34	Liquiritin	C21 H22 O9	418.1256	27.757	70.9	3.21E+08
35	(-)-Fustin	C15 H12 O6	288.063	23.386	74.8	1.93E+08
36	Pinocembrin	C15 H12 O4	256.073	27.77	80	8.96E+07
	flavan-3-ols					
37	Epigallocatechin	C15 H14 O7	306.0737	22.516	95.2	5.77E+08
38	Catechin	C15 H14 O6	290.0787	22.963	94.6	5.21E+08
39	(+)-Catechin hydrate	C15 H14 O6	290.0783	27.789	77.9	8.51E+07
	Biflavonoids					
40	Ginkgetin	C32 H22 O10	566.121	39.272	91.2	2.81E+07

41	Amentoflavone	C30 H18 O10	538.0896	33.187	86.2	2.71E+08
42	Bilobetin	C31 H20 O10	552.1051	35.228	85.3	5.63E+08
43	Sciadopitysin	C33 H24 O10	580.1366	43.554	81.1	3.65E+07
	Terpenoids					
44	Ginkgolide A	C20 H24 O9	408.1415	29.126	84.4	1.50E+10
45	Ginkgolide B	C20 H24 O10	424.1365	29.142	92.9	1.00E+10
46	Ginkgolide C	C20 H24 O11	440.1315	25.976	89.5	3.42E+09
47	Ginkgolide J	C20 H24 O10	424.1365	25.837	88.2	7.40E+08
48	Ginkgolide K	C20 H22 O9	406.126	33.187	92.2	3.32E+08
49	Bilobalide	C15 H18 O8	326.0996	26.336	86.5	1.12E+09
	Alkylphenolic acids					
50	Ginkgolic Acid C15:1	C22 H34 O3	346.2505	50.637	86.6	7.15E+06
51	Ginkgolic acid C17-1	C24 H38 O3	374.2819	52.660	79.5	3.10E+06
	Carboxylic acids					
52	Vanillic acid	C8 H8 O4	168.0421	21.672	80.4	7.19E+06
53	p-Coumaric acid	C9 H8 O3	164.0471	28.794	53.4	1.97E+07
54	Gallic acid	C7 H6 O5	170.0214	8.560	88.4	5.24E+07
55	3,4-Dimethylbenzoic acid	C9 H10 O2	150.0679	24.908	84.4	6.80E+07
56	2,3-Dihydroxybenzoic acid	C7 H6 O4	154.0265	18.996	98.7	5.25E+08
57	3-Coumaric acid	C9 H8 O3	164.0472	25.033	95.9	81054435
58	4-Coumaric acid	C9 H8 O3	164.0471	27.757	86.7	2.89E+08
	Lignans					
59	Lariciresinol 4-O-glucoside	C26 H34 O11	522.2096	24.939	85.5	2.75E+09
60	Pinoresinol diglucoside	C32 H42 O16	682.2471	23.564	90.7	5.95E+08
61	Dehydrodiisoeugenol	C20 H22 O4	326.1512	24.38	80.6	2.27E+08

				0		
62	Cycloolivil	C20 H24 O7	376.152	24.205	85.7	8.22E+07

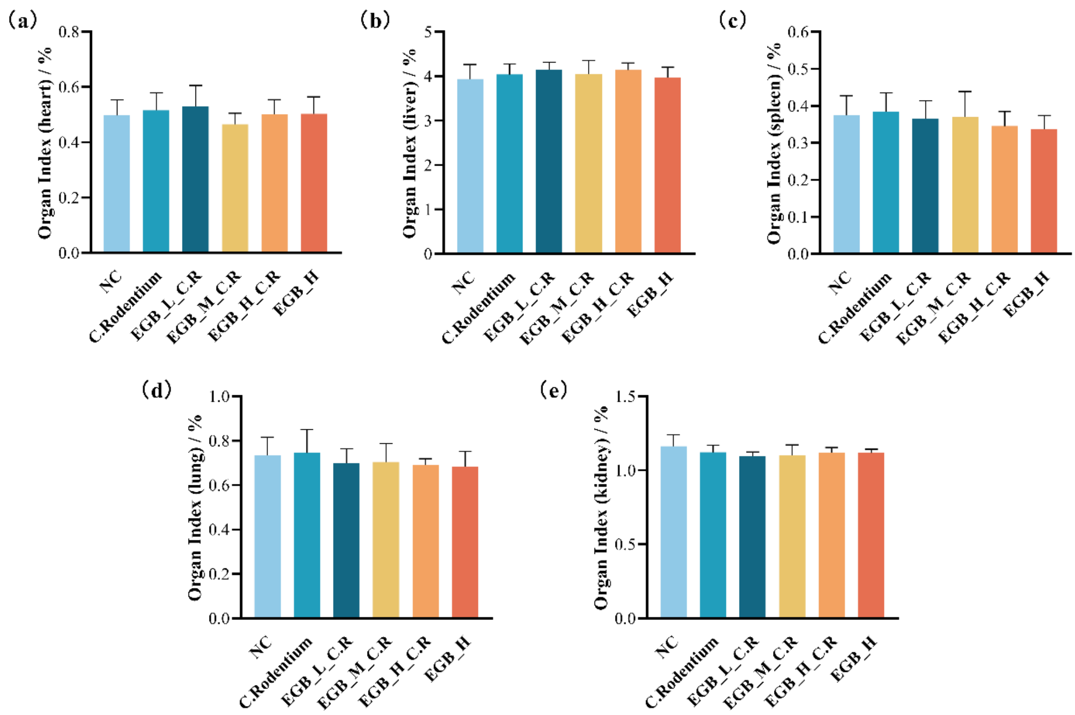

Figure S1. The organ coefficients of heart (a), liver (b), spleen (c), lung (d), and kidney (e) in all groups.

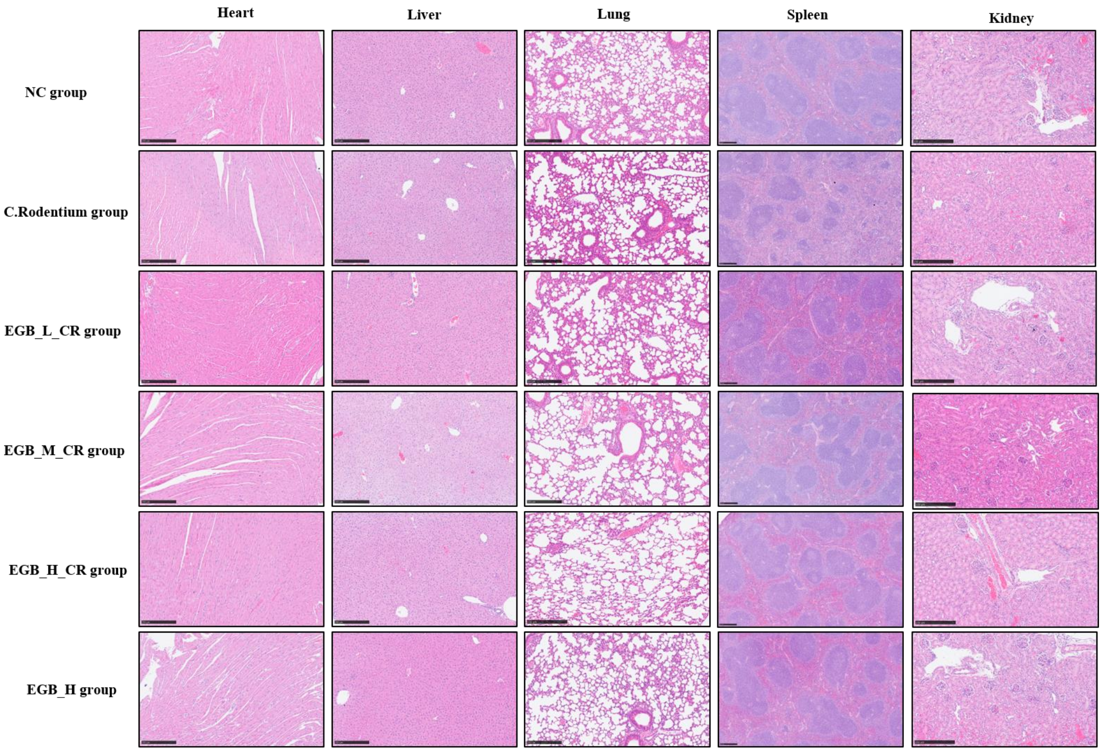

Figure S2. The histological evaluation of heart, liver, spleen, lung and kidney in all groups. All the scale bars are 250 μm.
